# Regional variability in therapeutic hypothermia eligibility criteria for neonatal hypoxic-ischemic encephalopathy

**DOI:** 10.1038/s41390-024-03184-6

**Published:** 2024-04-22

**Authors:** Jacopo Proietti, Geraldine B. Boylan, Brian H. Walsh

**Affiliations:** 1 INFANT Research Centre, University College Cork, Cork, Ireland; 2 Department of Paediatrics and Child Health, University College Cork, Cork, Ireland; 3 Department of Engineering for Innovation Medicine, Innovation Biomedicine section, University of Verona, Verona, Italy

## Abstract

**Abstract:**

Early induced therapeutic hypothermia represents the cornerstone treatment in neonates with probable hypoxic-ischemic encephalopathy. The selection of patients for treatment usually involves meeting criteria indicating evidence of perinatal hypoxia-ischemia and the presence of moderate or severe encephalopathy. In this review, we highlight the variability that exists between some of the different regional and national eligibility guidelines. Determining the potential presence of perinatal hypoxia-ischemia may require either one, two or three signs amongst history of acute perinatal event, prolonged resuscitation at delivery, abnormal blood gases and low Apgar score, with a range of cutoff values. Clinical neurological exams often define the severity of encephalopathy differently, with varying number of domains required for determining eligibility and blurred interpretation of findings assigned to different severity grades in different systems. The role of early electrophysiological assessment is weighted differently. A clinical implication is that infants may receive different care depending on the location in which they are born. This could also impact epidemiological data, as inference of rates of moderate-severe encephalopathy based on therapeutic hypothermia rates are misleading and influenced by different eligibility methods used. We would advocate that a universally endorsed single severity staging of encephalopathy is vital for standardizing management and neonatal outcome.

**Impact:**

- Variability exists between regional and national therapeutic hypothermia eligibility guidelines for neonates with probable hypoxic-ischemic encephalopathy.
- Differences are common in both criteria indicating perinatal hypoxia-ischemia and criteria defining moderate or severe encephalopathy. The role of early electrophysiological assessment is also weighted unequally.
- This reflects in different individual care and impacts research data. A universally endorsed single severity staging of encephalopathy would be crucial for standardizing management.

## Introduction

Hypoxic-ischemic encephalopathy (HIE) is a subtype of neonatal encephalopathy and a major contributor to global neonatal morbidity and mortality.^1^ It is caused by an intrapartum or perinatal event leading to reduced cerebral perfusion with insufficient supply of oxygen and glucose. This primary insult triggers a cascade of events which after a latent phase of approximately six hours, lead to a secondary deterioration characterized by oxidative stress, mitochondrial failure, neuroinflammation and extensive cell death.^2,3^

Therapeutic hypothermia (TH) represents the only proven treatment available to attenuate brain injury in infants with probable HIE, and current practice has shown its efficacy in reducing death and improving neurodevelopmental outcomes in survivors.^4^ TH should be instigated within six hours of birth, in the latent phase representing the window of opportunity to prevent the secondary programmed cell death. At present, the International Liaison Committee of Resuscitation only recommend it’s use for term or near-term neonates with moderate or severe encephalopathy,^5^ and the neuroprotective effect is obtained through 72 h of cooling (Whole body or Selective Head).^6^

The selection of patients for treatment usually involves meeting criteria indicating evidence of likely perinatal hypoxia-ischemia (HI) and the presence of significant neonatal encephalopathy on clinical examination. Neurophysiological monitoring with either amplitude integrated or conventional electroencephalography may also be used to assist this assessment. The eligibility criteria for cooling these infants are derived from previous randomized control trials (RCTs).^7^ Several guidelines on TH eligibility have been developed in an attempt to standardize care, however despite these protocols, there remains variability in practice.^8^ Even in jurisdictions with a published national TH eligibility protocol, significant between-unit variation in application and adherence to these protocols have been reported, resulting in differences in associated short-term outcomes.^9^

While adherence to protocols may be challenging, what is more concerning is differences between eligibility criteria themselves. Each of the TH RCTs differed slightly in their inclusion criteria. These minor differences in study inclusion criteria have resulted in alternate evidence based eligibility criteria being developed and implemented in routine care. Such variability between TH eligibility protocols is concerning and may be associated with risks. We have previously demonstrated that there are significant differences in TH eligibility depending on which evidence based guideline and exam criteria are used for infant assessment.^10,11^ Therefore, differences in TH eligibility guidelines and protocols can result in variation in whether an infant is eligible for neuroprotective therapy according to the location in which they are born, and differences in grade of encephalopathy assigned. Beyond the real impact of this on the individual infant, such variability also impacts the validity of data to assess between unit differences and track national trends of NE severity and TH eligibility.

In this review, we highlight the variability that exists between some of the different regional and national eligibility guidelines for TH, outlining differences in criteria indicating likely perinatal hypoxia-ischemia and criteria defining moderate or severe encephalopathy.

The guidelines that we reference are not an exhaustive list, rather we present a sample of readily available, frequently cited and recently published guidelines that represent a wide range of geographic regions, including national and regional protocols in use in Europe, North America, South America, Asia and Australia (listed in Supplementary Table 1).^12–22^

## Gestational age, birth weight and age following delivery

Each of the guidelines reviewed identified a minimum gestational age (GA) for TH eligibility, which ranged between 35 weeks and 36 weeks. Notably, only two of the TH RCTs included infants between 35 and 36 weeks GA, with 7 infants at 35 weeks GA randomized in total.^23,24^ In addition to gestational age, a minimum weight threshold of 1800 g was consistently identified in the various guidelines reviewed. These criteria were frequently used within the RCTs to limit the potential for confounding variables to impact the outcome, and concerns regarding potential variability in the safety profile. However it should be noted that variation in clinical practice is emerging, with single centers reporting their local practices include offering TH to infants down to 34 weeks PMA.^25,26^ However there remains no evidence for efficacy, with preliminary data from the Preemie Hypothermia Trial reporting no benefit in cooling infants between 33 and 35 + 6 weeks GA.^27^

All of the reviewed guidelines recommend that TH is instigated within 6 h after birth. This is supported by data from the TH RCTs.^24,28^ The evidence for the potential benefit of TH started after 6 h of life is controversial.^29,30^ The late hypothermia trial by Laptook et al. reported that if TH was initiated between 6 and 24 h of age, there was a 76% chance of at least a 1% improvement in death or disability at 18–24 months.^30^ While this is statistically significant, the clinical significance is less clear. However, while advocating for initiating TH within the first 6 h, most guidelines envisage the possibility of initiating treatment later i.e., up to 12 or 24 h where infants are identified after 6 h.^14,16^

## Criteria indicating evidence of acute perinatal hypoxia/ischemia

The criteria indicating evidence of acute perinatal/intrapartum hypoxia-ischemia (HI) differ between published guidelines. Examples are shown in Table 1. This is reflected in the Cochrane review of Therapeutic hypothermia. Among the criteria for studies to be included in the Cochrane review was that the RCT’s definition of perinatal asphyxia had to include at least one of the following; Apgar score ≤5 at 10 min, or; cord pH ≤ 7.1, or an arterial pH ≤ 7.1 or BD ≥ 12 in first hour of life, or; mechanical ventilation or resuscitation at 10 min. However reflecting the variability in studies the actual inclusion criteria of the RCTs entered into the systematic review could be much broader (or narrower) than this, as they only needed to meet one of these criteria. As such the Cochrane review itself does not advocate for any particular defining thresholds of perinatal asphyxia to meet TH eligibility criteria.^31^

**Table 1:** Examples of criteria indicating evidence of acute perinatal hypoxia-ischemia.

	North America		South America	Europe			Asia	Australia and New Zealand		
	NICHD-PRIME	Canada	SIBEN	Netherlands NVK	UK-BAPM	Ireland	Japan	Queensland	Sydney NETS	New Zealand NZCYCN
Gestational age (weeks)	≥36	≥36 (note on ≥35)	>35	≥35	≥36	≥36	≥36	≥35	≥35	≥35
Postnatal age (hours)	≤6	≤6	Not mentioned	<6	≤6	≤6	≤6	<6	<6	<6
Minimum weight (kg)	>1,8	Not mentioned	Not mentioned	>1,8	Not mentioned	≥1,8	≥1,8	≥1,8	Not mentioned	≥1,8
Evidence of acute intrapartum/perinatal hypoxia-ischaemia	pH ≤ 7.00 or BD ≥ 16 on any cord or baby gas within 1 h OR pH 7.01 to 7.15 or BE 10 to 15.9 on any cord or baby gas within 1 h plus both the following: - history of acute perinatal event - Apgar ≤5 at 10’ or assisted ventilation initiated at birth and continued for at least 10’	Cord pH ≤7.0 or BD ≥ 16 OR pH 7.01 to 7.15 or BE 10 to 15.9 on any cord or baby gas within 1 h plus both the following: - history of acute perinatal event - Apgar ≤5 at 10’ or requirement for positive-pressure ventilation at 10’	History of acute perinatal event, associated with low Apgar (≤5 at 1’, 5’ or 10’) or pH ≤7.1 on cord gas Not mandatory: “If the clinical score demonstrates clear signs of encephalopathy, this should not be ignored even with a pH > 7.1”	≥1 of the following: - Apgar ≤ 5 at 5’ - resuscitation at birth - requirement for respiratory support for ≥10’ - pH < 7.0* - BD > 16 mmol/L* - lactate >10 mmol/L* *on any cord or baby gas within 1 h	≥1 of the following: - Apgar ≤ 5 at 10’ - continued need for resuscitation at 10’ - pH ≤ 7.0 or BD ≥ 16 on any cord or baby gas within 1 h	≥1 of the following: - pH <7.0 or BD ≥ 16 on any cord or baby gas within 1 h - Apgar ≤5 at 10’ - continued need for PPV or intubated at 10’	≥1 of the following: - Apgar ≤5 at 10’ - continued need for resuscitation at 10’ - pH < 7.0 or BD ≥ 16 on any baby gas within 1 h	≥1 of the following: - Apgar ≤5 at 10’ - pH <7.0 or BD ≥ 12 on any cord or baby gas within 1 h -mechanical ventilation or ongoing resuscitation for ≥10’	≥1 of the following: - pH <7.0 or BD ≥ 12 on any cord or baby gas within 1 h - Apgar ≤ 5 at 10’ - Mechanical ventilation or ongoing resuscitation for ≥ 10’ - pH 7–7.1 or lactate >8 mmol/L in the first hour	≥1 of the following: - Apgar ≤ 7 at 10’ - mechanical ventilation >5’ or ongoing resuscitation for ≥10’ - pH <7.1 or BD ≥ 12 or lactate >6 on any cord or baby gas within 1 h

Depending on individual guidelines, determining the potential presence of perinatal HI may require either one, two or three signs among the following categories: abnormal blood gases, history of acute perinatal event, low Apgar score, prolonged resuscitation at delivery.

All of the reviewed guidelines included a blood gas as a key data point when assessing for evidence of the presence of perinatal HI. In some guidelines^12,14,18^ the blood gas is weighted more heavily compared to other criteria of potential perinatal HI, and may be the only criteria required to indicate the presence of HI. In most guidelines, acidosis is defined as the alteration of either pH or BD; in some only pH is considered.^15^ A range in cut-off values are used as entry criteria for TH therapy in the published guidelines, from ≤7.0 to ≤7.15 for pH, and >12 to ≥16 mmol/L for BD. The lactate level is seldom included as an indicator for eligibility, but in those guidelines that did include it, the threshold cut-off for eligibility ranged from 6 to 10 mmol/L.^16,21,22^ The suggested source of blood gas is cord gas or any baby blood gas (arterial, venous, or capillary) within one hour of birth in most guidelines. Therefore, for pragmatic reasons, the differences in acid-base levels between arterial and venous blood samples is not considered when determining threshold values for TH eligibility in the published guidelines.

A history of a clearly recognized perinatal event is sometimes included, although never a mandatory element for the definition of perinatal HI.^12,14,15,18^ Fetal heart rate decelerations, cord prolapse or rupture, placental abruption, uterine rupture, maternal trauma or hemorrhage are variably included when defining evidence of potential perinatal HI.

All guidelines include a low Apgar score as potential evidence of an acute perinatal HI event. Despite this, there is less agreement on actual scores or timing of scores. The cut-off for an Apgar score indicating depression at birth varied from <5 to ≤5 in the majority, but was as high as ≤7 in one guideline.^22^ Similarly, while most guidelines only included the 10-min. Apgar, one used the 5 min. Apgar score,^16^ and an alternate included an Apgar score of ≤5 at any time point, 1, 5 or 10 minutes.^15^ Continued need for resuscitation and/or ventilation for 10 or more minutes after birth, is also considered as evidence of potential perinatal HI, and is included in most guidelines.

Therefore it is clear that while there is good agreement on what criteria are relevant for screening assessment, there remains tangible differences between the published guidelines in several of the domains that are assessed.

## Criteria representing the presence of encephalopathy or defining moderate or severe encephalopthy

The neurological exam has a critical role in determining eligibility for TH. HIE is traditionally classified in stages, which if applied consistently provide useful information about the severity of injury. All guidelines reviewed aimed to identify neonates with moderate or severe encephalopathy. A variety of clinical scoring schemes for HIE have been developed, without universal agreement on which is most accurate. Most guidelines however rely on neurological scoring systems adapted from the seminal work of Sarnat and Sarnat.^13,14,18,21,32^ However there is some variation with the Dutch guidelines being based on the Thompson score,^16^ and the British association of perinatal medicine (BAPM) guidelines using the neurological abnormality entry criteria from the TOBY trial.^17^ The authors are unaware of any TH RCTs using the Thompson score for determining TH eligibility, however it was used In the neo.nEURO.network RCT^33^ to evaluate the neurological status at 7 days of age, and the association between Thompson score and the different Sarnat stages has been well documented previously.^34^

Regarding the original system proposed by Sarnat and Sarnat it is worth noting that it was meant as a prognostic test based on serial evaluations over the first week, at a time when no early intervention was available. It was never intended to be a single point test for determining the severity of encephalopathy in the first 6 h after birth. Sarnat et al. have recently published a Commentary proposing to update the Sarnat exam, however the detailed protocol to address changes is yet to be published.^35^ Therefore it is unclear at this time if any update would impact current definitions for severity of encephalopathy.

In general, the following domains are assessed in newborn neurological examinations assessing TH eligibility (examples in Table 2): level of consciousness, spontaneous activity, posture, tone, primitive reflexes and autonomic activity. Most guidelines reviewed included all of these domains, however there was some variation. Spontaneous activity is not included in the Dutch, British and Japanese guidelines, while posture is not included in the British, Japanese and New Zealand guidelines.

**Table 2:**
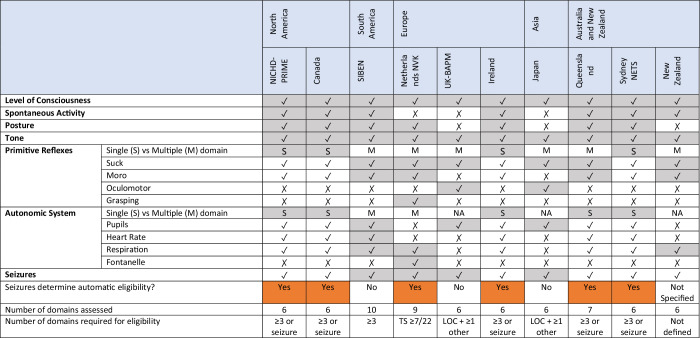
Examples of domains assessed in the clinical neurological exam.

The primitive reflex domain includes single reflexes assessed independently: suck, Moro (not included in the British and Japanese guidelines), oculomotor (only included in the British and Japanese guidelines^19^) and grasp (only included in the Dutch guidelines^16^). In some guidelines each reflex represents a category, with the same importance as every other single main domain.^15–17,19,20^ In others, individual reflexes represent subdomains, with the worst score providing the global grade for the overall primitive reflex domain.^13,14,18,21^

Similarly, the autonomic system domain variably includes the evaluation of pupils, heart rate, respiration and fontanelle. In the SIBEN and Dutch guidelines each of the latter features represents a category, with the same importance as every other single domain.^15,16^ In the British, Japanese and New Zealand guidelines, one single autonomic parameter is examined.^17,19,22^ In other guidelines they represent subdomains and similar to the primitive reflex domain, the worst score across any autonomic system sub-domain provides the overall domains’ score.^13,14,18,21^

In many guidelines the neurological evaluation schemes allow the distinction between mild, moderate, and severe encephalopathic features for all the domains examined, and result in a diagnosis of mild, moderate, or severe encephalopathy. However in some, features consistent with either normal or mildly abnormal are grouped together,^18^ and in others only features consistent with moderate and severe encephalopathy are included.^14^ Of note, based upon the TOBY RCT, the British and Japanese guidelines eligibility criteria do not identify a related grade of severity- only if the child is eligible or not.^17,19^

When determining if an infant is eligible for TH or not, most guidelines indicate that if 3 or more domains are consistent with moderate or severe encephalopathy then the infant is eligible for TH. The Dutch guidelines use a cut-off of 7 points (on a total of 22) on the Thompson score to determine clinical eligibility, regardless of the severity of the scores given in the single domains.^16^ The British and Japanese criteria both give more weight to the level of consciousness over the other exam components, and require an abnormal level of consciousness plus an abnormality in one further domain to determine TH eligibility.^17,19^

In addition to these differences, there is variation in the interpretation of specific findings for individual criteria that are shared between guidelines, as summarized in Table 3. Decreased spontaneous activity may be characterized as a mild or moderate grade in the NICHD guidelines,^13^ while it is invariably classified as moderate in other guidelines.^14,15,18,20–22^ Regarding tone, both hypotonia and hypertonia are defined as moderate abnormalities in NICHD guidelines whereas in the other systems, only a reduced tone is considered a moderate finding, while having increased tone is often classified as mild.^16,18,20,22^ For posture, the interpretation of distal flexion of the limbs is particularly controversial: some guidelines interpret any distal flection as a moderately abnormal posture,^14,18^ while others distinguish between mild and moderate distal flexion, with each being interpreted as a marker of mild or moderate encephalopathy respectively.^13,15,16,21^ For the primitive reflexes, weak suck and Moro are considered moderately abnormal findings indicating eligibility in some guidelines,^14,18,20^ while in others they are also classified as mild.^16^

**Table 3:**
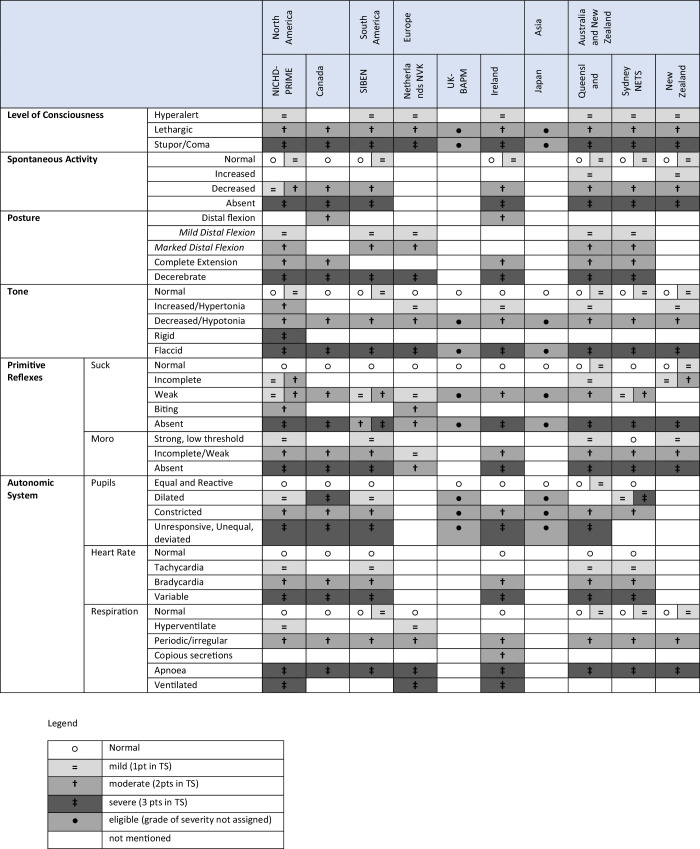
Examples of severity interpretation of specific findings in neurological clinical exam.

These differences in the interpretation and criteria used to identify the presence of moderate and severe encephalopathy lead to real world differences in the selection of patients for treatment. A clinical research study comparing eligibility using NICHD and British criteria revealed a significant difference in the proportion of infants determined to be eligible for TH depending on which exam is used, with 24% more infants being eligible for the NICHD but ineligible for the British criteria. Interestingly, in that study more than a half of infants in which a discrepancy of eligibility was found demonstrated MRI evidence of cerebral injury, while neither method identified all infants who developed seizures or had cerebral MR injury.^11^ Another comparison study between NICHD and SIBEN grading systems, despite a good agreement between methods (92%), highlighted that SIBEN defines significantly more infants as moderate and less as mild, than NICHD. In the same study, numerical scores were also assigned using the same methods, and proved to be superior to standard grades in defining a minimum threshold for cerebral injury.^10^

## Time of evaluation

Neonatal encephalopathy is a dynamic process, and the severity of neurological findings often change over time. To ensure prompt treatment, it is critical to define the severity of encephalopathy within the narrow window of time available to initiate treatment.

A minimum age at which the neurological examination is reliable in detecting encephalopathy has not been defined. Animal studies suggest that the earlier the treatment is started during the latent phase the better it is in preventing secondary injury in the brain and improving outcomes.^36^ In humans, the TOBY trial demonstrated a trend towards better outcomes in infants in which TH was initiated in the first 4 h after birth^37^; and Thoresen et al. described an improved motor outcome in a cohort of newborns cooled within 3 h.^38^ Following this, some regional guidelines advise that the neurological examination should be done as soon as possible after the baby is stabilized and within the first hour of birth.^15,22^ However other guidelines recommend assessing the neurological criteria only after 1 h and before 6 h after birth.^18,20^ There is a lack of evidence about which approach is more appropriate, as the RCTs did not specify a minimum age of evaluation for study entry.^24,28^ Nonetheless, an exam performed immediately post resuscitation is potentially not a true reflection of the neurological status, and waiting for an hour after birth (allowing the baby to recover from the initial resuscitation) appears to be a pragmatic decision.

The majority of guidelines indicate repeated frequent (hourly) assessment of neurological status within the first 6 h of birth.^14–16,20,21^ Babies who meet any of the criteria for significant perinatal HI but on initial neurological examination are neurologically normal or mild, should be reviewed several times in order to capture a possible evolution and deterioration of the exam within the first 6 h. On the other hand, two guidelines^14,16^ clearly state that a neonate with a neurological exam that initially meets eligibility criteria, but that rapidly improves within the first few hours may not need TH. This practice is at odds with the concept that the benefit of TH is greater the earlier that it is initiated, and while there is some evidence for early exit from TH at 24 h of age in low risk infants,^39^ there is no evidence to support or refute this practice in the first 6 h.

## Seizures

The presence or absence of seizures is included in all the guidelines examined. In some guidelines, evidence of seizures in the first 6 h is included similar to each domain of the neuro exam, and therefore it is possible to have seizures but not meet threshold for TH.^15,17,19^ Contrary to this, in other guidelines, seizures in the first six hours among infants with evidence of perinatal HI represent an independent indication for TH, even when infants do not have sufficient additional moderate or severe criteria to meet standard TH threshold based on neurological examination.^12,14,16,18,20,21^ In other words, if a patient is less than 6 h old and meets the gestation, weight and blood gas criteria and has a witnessed seizure, the patient is eligible for TH regardless of neurological examination findings.

However, the level of diagnostic certainty of seizures is rarely specified in TH eligibility guidelines. The SIBEN paper refers to seizures assessed clinically, while regional guidelines from Australia refer to seizures witnessed by the medical officer, nurse, or midwife.^20,21^ EEG-confirmation is rarely deemed to be necessary. Nevertheless, it is now well known that diagnosis of neonatal seizures based on clinical exam is difficult and often inaccurate, regardless of the level of experience of the clinician. Seizure like movements are often misinterpreted and only clonic seizures can be reliably diagnosed based on clinical evaluation.^40,41^ In the case of suspected seizures in the immediate post-natal period, the clinician should seek EEG/aEEG confirmation when possible.

In addition, in the event of acute provoked seizures emerging within the earliest 6 h after birth, a subacute injury evolving during the course of labor should be suspected. Literature on the temporal characteristic of seizures in neonatal HIE has flourished in recent years, and the median age at electrographic seizure onset in HIE falls beyond 12 h of age in most studies. Seizures occurring before 6 h of age are rare in an acute perinatal HI event.^42^ In essence, neonatal acute provoked seizures are a symptom of ongoing encephalopathy, but when detected in the earliest hours after birth they are likely to be an expression of a subacute injury which started and evolved in the hours before birth. If this is the case, even early treatment may partially miss the window of opportunity for successful intervention.^43^ Nonetheless, in clinical practice, given that the exact timing of injury will rarely be identified, it is reasonable and appropriate to cool neonates with evidence of perinatal HI and witnessed seizures in the first 6 h. In fact, these neonates were included in the RCTs and a decision not to cool would represent a deviation from the current evidence base. Furthermore, since there is evidence that TH reduces seizure burden^42,44^ which in turn may affect neurodevelopmental outcome, treatment instigated beyond the optimal therapeutic window could still potentially reduce the on-going injury.

## Role of aEEG

Use of amplitude integrated EEG to supplement the early assessment is encouraged in some, but not in the majority of guidelines.^14,22^ The BAPM guidelines do state that infants who meet exam criteria should then have an aEEG performed, however even then it is stressed that if both perinatal HI and clinical encephalopathy criteria are met that initiation of TH should not be delayed if an aEEG is not promptly available.^17^ The recommended duration of the aEEG monitoring varies from 20 to 30 min, and the altered aEEG patterns recognized as supporting or determining treatment eligibility are slightly different between the various guidelines. The definition of these patterns is more detailed in the Dutch guidelines: discontinuous normal voltage (with lower limit equal to or lower than 5 μV), discontinuous low voltage (periods of very low voltage interspersed with peaks of high amplitude), continuous low voltage (constantly around or lower 5 μV) and flat trace (deeply depressed activity near to isoelectric) all indicate TH eligibility.^16^ The other guidelines mention the presence of an abnormal baseline or moderately abnormal activity, discontinuity or suppressed activity, but do not further define these criteria. The identification of seizures on the aEEG is invariably described as an indication for TH, and regarding this we refer to the discussion in the previous paragraph.

In most guidelines aEEG findings represent a supporting criterion, and play a subordinate role to clinical assessment in the selection for treatment. Discrepancy between the clinical grade and the aEEG severity may occur. In the CoolCap trial, 8 neonates classified as mildly encephalopathic based on clinical evaluation showed moderately or severely abnormal patterns on the initial aEEG evaluation.^45^ In a more recent cohort study, 13 infants were reported to have moderately abnormal aEEG findings despite a mild clinical exam; 31% later displayed an abnormal MRI.^46^ Some of the existing guidelines advocate that if there is a discrepancy between findings on aEEG and neurological examination a decision should be made based on physical examination findings.^19^ Contrary to this the Dutch guidelines consider the aEEG as non-inferior to the clinical neurological assessment for TH eligibility: an abnormal aEEG can indicate treatment eligibility even in the absence of the clinical criterion of a Thompson score >7.^16^ This approach is supported by recent work from the Netherlands which showed; (1) that the aEEG and Thompson score in the first 6 h were equally predictive of long-term outcome^47^; and (2) some infants who were found to have a low Thompson score, but an abnormal aEEG assessment <6 h of age, ultimately had a poor long-term outcome.^48^ The authors concluded that while they could not determine if one method is superior to the other, using the aEEG helped to identify cases for TH that would not have been offered treatment based on clinical exam alone.^47^

Formal EEG was included in the original Sarnat staging system, and the valuable real time information provided by both aEEG and EEG should be thoughtfully considered when available. A recent study focused on neonates with HIE clinically defined as mildly encephalopathic in the first 6 h after birth, demonstrated a wide spectrum of electrographic dysfunction on multichannel EEG. One third of infants monitored had moderate to severely abnormal background EEG patterns, which were associated with a higher risk of developing acute provoked seizures. Contrary to this, those infants that were clinically mild and who had a normal or mildly abnormal early EEG background were at lower risk for acute provoked seizures.^49^ Therefore it is clear that neurophysiological monitoring (aEEG or EEG), can provide additional information to the clinical exam,^48^ and if moderate or severely abnormal may assist in determining need for TH. However it must be recognized that access to neurophysiological monitoring and the expertize required to interpret them is frequently not available (e.g., in smaller units and during transport). It is hoped that in the future the development of mobile devices providing real-time aEEG/EEG monitoring with automated or centralized review, will make neurophysiological monitoring more widely available in all health care settings.^50^

## Milder encephalopathy

For infants with probable HIE, the category of mild encephalopathy is often controversial. There are no consensus recommendations on which of these infants should be monitored with aEEG/EEG, who should receive neuro-imaging, or even how long they should be followed post-discharge. Most controversial of all however is how best to manage them. Many infants with milder encephalopathy are being treated on clinical judgment, without fulfilling eligibility criteria defined in the guidelines. Mehta and collaborators, in a retrospective study on TH infants born between 2007 and 2011 in New South Wales and Australian capital territory, found that 50% did not meet regional eligibility criteria, and 70% did not fulfill the criteria for “evidence of asphyxia”.^51^ Data from TH registries set-up in Europe and in the United States after completion of the TH RCTs revealed that around 40% of infants receiving TH lacked clinical features of moderate or severe encephalopathy.^52,53^ This data is over a decade old now, and in the interim the use of TH in mild encephalopathy has been increasing internationally.^54–56^ This practice is driven by concern that these infants, historically considered at minimal risk for adverse outcomes,^57^ are at risk of injury. The evidence of injury among mild encephalopathy is now well recognized, with recent studies demonstrating significant risk of cerebral injury and adverse neurodevelopmental outcomes in this population.^58–61^ Nonetheless, there is minimal to no data on the risk profile associated with TH in mild HIE^24^; stress related to the exposure to hypothermia, sedative administration, delay in the initiation of feeds, separation from parents, and longer hospital stay are all elements that deserve to be considered. The balance of risk against potential benefit is unknown and where best to draw that line in the care of mildly encephalopathic neonates is the subject of active debate and research. Additionally, the scoring systems incorporated in currently used treatment guidelines are focused on identification of moderate and severe encephalopathy and there is no consensus on the accurate definition of mild encephalopathy within the first 6 h after birth.^62^ In many centers for children with probable HIE, mild is defined as encephalopathy not meeting local guidelines for TH eligibility, in essence a diagnosis of exclusion. Given the variability in guidelines discussed here, such a definition for mild encephalopathy is fraught with issues. Furthermore, the current method of dividing severity of encephalopathy into three grades is probably an over simplification of the clinical spectrum that exists. Numerical scoring systems based on Sarnat, Thompson and SIBEN scores have been recently proposed.^10,13,62^ These systems, which acknowledge the wide spectrum associated with encephalopathy may be better suited to demonstrate and detect the range of clinical variability. Studies prospectively validating such scores in terms of outcomes, identified threshold NICHD Total Sarnat Score of ≥5 or ≥4 (representing infants at the sicker end of mild encephalopathy) as providing the best sensitivity for identifying neonates who would have neurodevelopmental issues, highlighting in particular those at greater risk within the mild encephalopathy group (who fall outside of the classical TH eligibility).^10,13^

## Conclusions

Remarkable differences emerge when comparing TH eligibility criteria in different jurisdictions. Most systems require infants to demonstrate evidence of perinatal hypoxia-ischemia plus clinical findings consistent with moderate to severe encephalopathy using a standardized exam. However the criteria indicating evidence of acute perinatal hypoxia-ischemia are not uniform between different guidelines, nor is the clinical neurological examination used to confirm the eligibility. These evidence based exams often define the severity of encephalopathy differently, with varying number of domains required for determining eligibility and blurred interpretation of findings assigned to different severity grades in different systems. aEEG is commonly used to support clinical decision making, but in many centers it is not available in the first hours after birth and its specific importance varies between guidelines and countries.

Due to the lack of clear agreed definitions for criteria indicating perinatal hypoxia-ischemia and moderate to severe encephalopathy, an individual infant’s eligibility status for TH differs between centers and nations. A clinical practice implication is that infants may receive different care depending on the location in which they are born. This could also impact epidemiological data, as inference of rates of moderate-severe encephalopathy based on TH rates are misleading and influenced by different eligibility methods used. We would advocate that a universally endorsed single severity staging of encephalopathy is vital for standardizing management and neonatal outcome. The NICHD expanded scoring system, and the associated Total Sarnat Score, are the most frequently referenced for research studies, implying a greater familiarity for clinicians, which would ease adoption across sites and nations. However we would additionally advocate for the incorporation of additional neurophysiological monitoring (aEEG/EEG) into the initial assessment when available, to supplement and support the clinical exam.

## Supplementary information

Supplementary Material
