# Supplementary material for: Regional variability in therapeutic hypothermia eligibility criteria for neonatal hypoxic-ischemic encephalopathy
Source: Pediatr Res. 2024 Apr 22;96(5):1153–61. doi: 10.1038/s41390-024-03184-6 (PMC11521984; doi:10.1038/s41390-024-03184-6)
Supplement: Supplementary file 1 — Supplementary Material [file 41390_2024_3184_MOESM1_ESM.pdf]

Supplementary table 1: List of national and regional protocols used in this review.

Shankaran S, Laptook AR, Ehrenkranz RA, Tyson JE, McDonald SA, Donovan EF, Fanaroff AA, Poole WK, Wright LL, Higgins RD, Finer NN, Carlo WA, Duara S, Oh W, Cotten CM, Stevenson DK, Stoll BJ, Lemons JA, Guillet R, Jobe AH; National Institute of Child Health and Human Development Neonatal Research Network. Whole-body hypothermia for neonates with hypoxic-ischemic encephalopathy. N Engl J Med. 2005 Oct 13;353(15):1574-84. doi: 10.1056/NEJMcps050929.	NICHD RCT	Shankaran et al., 2005
Chalak LF, Adams-Huet B, Sant'Anna G. A Total Sarnat Score in Mild Hypoxic-ischemic Encephalopathy Can Detect Infants at Higher Risk of Disability. J Pediatr. 2019 Nov;214:217-221.e1. doi: 10.1016/j.jpeds.2019.06.026.	PRIME (NICHD expanded scoring system)	Chalak et al., 2019
Lemyre B, Chau V. Hypothermia for newborns with hypoxic-ischemic encephalopathy. Paediatr Child Health. 2018 Jul;23(4):285-291. doi: 10.1093/pch/pxy028.	Canadian	Lemyre and Chau, 2018
Perez JM, Golombek SG, Sola A. Clinical hypoxic-ischemic encephalopathy score of the Iberoamerican Society of Neonatology (Siben): A new proposal for diagnosis and management. Rev Assoc Med Bras (1992). 2017 Jan 1;63(1):64-69. doi: 10.1590/1806-9282.63.01.64.	SIBEN	Perez et al, 2017
Nederlandse Vereniging voor Kindergeneeskunde (NVK). Therapeutische hypothermie na perinatale asfyxie, versie 4.3, maart 2014 Herziene versie 5.11 – 2309 2021. Available at: Over de samenvatting (neonatology.eu)	Dutch	NVK, 2021
British Association of Perinatal Medicine (BAPM). Therapeutic Hypothermia for Neonatal Encephalopathy, a framework for practice. Available at: https://www.bapm.org/resources/237-therapeutic-hypothermia-for-neonatal-encephalopathy *(last modified 16 December 2020, accessed 09 August 2023).*	British	BAPM, 2020
San Lazaro Campillo I, McGinley J, Corcoran P, Meaney S, McKenna P, Filan P, Greene RA, Murphy J on behalf of Neonatal Therapeutic Hypothermia Steering Group. Neonatal Therapeutic Hypothermia in Ireland, Annual Report 2016-2020. Available at: https://www.hse.ie/eng/about/who/acute-hospitals-division/woman-infants/national-reports-on-womens-health/neonatal-therapeutic-hypothermia-in-ireland-annual-report-2020.pdf	Irish	San Lazaro Campillo et al., 2020
Takenouchi T, Iwata O, Nabetani M, Tamura M. Therapeutic hypothermia for neonatal encephalopathy: JSPNM & MHLW Japan Working Group Practice Guidelines Consensus Statement from the Working Group on Therapeutic Hypothermia for Neonatal Encephalopathy, Ministry of Health, Labor and Welfare (MHLW), Japan, and Japan Society for Perinatal and Neonatal Medicine (JSPNM). Brain Dev. 2012 Feb;34(2):165-70. doi: 10.1016/j.braindev.2011.06.009.	Japanese	Takenouchi et al., 2012
Queensland Clinical Guidelines. Hypoxic ischaemic encephalopathy (HIE). Guideline No. MN21.11-V11-R26. Queensland Health. 2021 Available from: http://www.health.qld.gov.au/qcg	Queensland	Queensland Clinical Guidelines, 2021
Hypoxic Ischaemic Encephalopathy in the Newborn. Sydney Children's Hospitals Network, Newborn and Paediatric Emergency Transport Service (NETS). Date of Publishing: 15 March 2022. Accessed: 21 September 2023.	Sydney	NETS, 2022
Neonatal Encephalopathy Consensus Statement from the Newborn Clinical Network. New Zealand Child and Youth Clinical Networks (NZCYCN). Date last published: 30 October 2019. Available from: Neonatal Encephalopathy Consensus Statement from the Newborn Clinical Network (starship.org.nz)	New Zaeland	NZCYCN, 2019
